# CAR‐T treatment: Determining the progression‐free survival gain in patients with heavily pretreated multiple myeloma

**DOI:** 10.1002/jha2.63

**Published:** 2020-07-13

**Authors:** Andrea Messori

**Affiliations:** ^1^ HTA Unit Regional Health Service Florence Italy

At the 2020 ASCO Meeting, important findings have been presented on the effectiveness of idecabtagene vicleucel (ie, CAR‐T by Bristol/Bluebird also denoted as ide‐cel‐bb2121) in heavily pretreated multiple myeloma patients. The outcomes in 128 patients given idecabtagene vicleucel in the KarMMa trial (dose: 150 to 450 × 10^6^ CAR+ T cells) were indirectly compared with those of 190 patients selected from a real‐world database of 1949 patients. The subgroup of 190 patients was identified through propensity matching to represent an adequate control group for the 128 patients of the KarMMa trial. All patients had received at least three previous lines of treatment. The endpoint was progression‐free survival (PFS). The median PFS was 11.3 months in the treatment group and 3.5 months in the controls [1].

In the last years, an extensive literature has accumulated on the use of restricted mean survival time (RMST) for the interpretation of survival curves [2, 3, 4, 5, 6, 7, 8, 9, 10]. In comparison with traditional analyses based on hazard ratio (HR) and medians, the RMST has important advantages because it examines the entire survival curve (like the HR) and expresses the survival outcomes using a scale of time (like medians). Most previous experiences on the application of RMST are focused on oncology [2, 3, 4, 5, 6, 7, 8]. Quite recently, the application of RMST has been investigated in the field of CAR‐T [11, 12]. Briefly, the RMST combines the main advantages of HR and medians without possessing their disadvantages.

From a practical point of view, the RMST is characterized by a high mathematical complexity of its statistical calculations [2, 3, 4, 5, 6, 7, 8, 9, 10]. However, recent papers have suggested an original method of calculation, drawn from the field of pharmacokinetics, that allows for an extreme simplification of RMST estimation. This new method [13, 14, 15], derived from pharmacokinetics, first requires to digitize the published graph of the Kaplan‐Meier curve [16]; this generates around 50‐100 data pairs of survival probability‐versus‐time (ie, *y*‐vs‐*x* data pairs); then, as in pharmacokinetics, the trapezoidal rule is applied to determine the area under the curve (AUC) using a simple Excel subroutine [17]. The AUC is known to be equal to the RMST.

In the present analysis, we employed the RMST to assess the PFS gain for heavily pretreated patients given idecabtagene vicleucel (experimental group) versus matched control patients of the real world (Figure 1). Our results based on the RMST were compared with those based on the medians originally reported in the study by Jagannath and co‐workers [1] (Table 1).

**TABLE 1:** Characteristics of the two cohorts and values of RMST and medians estimated from the two time‐to‐event curves. End point, progression‐free survival

						Survival gain (mos)	
Dataset	Treatment	t^*^ (mos)	No. of patients	RMST (mos) with 95% confidence interval	Median (mos) with 95% confidence interval	From RMST	From medians^b^
KarMMa trial	CAR‐T (idecabtagene vicleucel)	18	128	9.92^a^ (9.56 to 10.29)	11.3 (9.5 to 13.1)^b^	3.41^c^	7.80^d^
Matched controls	Various standards of care		190	6.51^a^ (6.22 to 6.80)	3.5 (3.2 to 3.7)^b^		

*Note*. The milestone is the time point in the follow‐up at which the area under the PFS curve is truncated; t^*^ was chosen as the longest follow‐up reached by both curves.

a Statistical testing (unpaired *t*‐test) between the two cohorts indicates a significant difference in favor of the CAR‐T group (*P* < .0001).

b Information as reported in the original trial.

c Difference of 9.92 minus 6.51 mos.

d Difference of 11.3 minus 3.5 mos (*P* < .0001 according to Jagannath and coworkers [1]).

Abbreviations: RMST, restricted mean survival time; t^*^, milestone employed in the RMST analysis; mos, months.

The main original finding expected from our indirect comparison was to estimate the PFS gain for CAR‐T compared with the controls according to the RMST methodology. Apart from its level of statistical significance, the extent of this PFS improvement in favor of CART‐T was quite small when assessed through the RMST (gain of 3.41 months at 18 months, ie, the difference of 7.80 minus 3.41 months; Table 1). In contrast, the gain estimated from the medians by Jagannath et al. [1] was much longer (7.80 months). The RMST in fact generates more stable and more reliable gains than the median because the median has a “punctiform” nature and, therefore, is strongly influenced by the small portion of follow‐up when residual survival goes from >50% to <50%.

Of course, beyond the milestone currently set at 18 months, the patients given CAR‐T might have, in future perspective, a longer additional PFS than those not given CAR‐T, but this hypothesis will need confirmation by studying the patients of the KarMMa trial on the long term. Because the information about the efficacy of CAR‐T in multiple myeloma is still very limited, the comparison presented herein is interesting because it is the only one that can presently be made.

Two conclusions are suggested by our analysis. First, the RMST is shown to be a suitable parameter for managing the PFS data of multiple myeloma patients receiving a CAR‐T. Second, the RMST analysis based on the outcomes currently available does not demonstrate any breakthrough PFS advantage for idecabtagene vicleucel in comparison with treatments not involving any gene manipulations.

In conclusion, when the RMST is employed as the outcome measure of the analysis, the relevance of the clinical advantage generated by CAR‐T in patients with myeloma seems to be more limited than that suggested by the medians and reported in the KarMMa trial [1].

## CONFLICT OF INTEREST

The author declares no conflict of interest.

**FIGURE 1:**
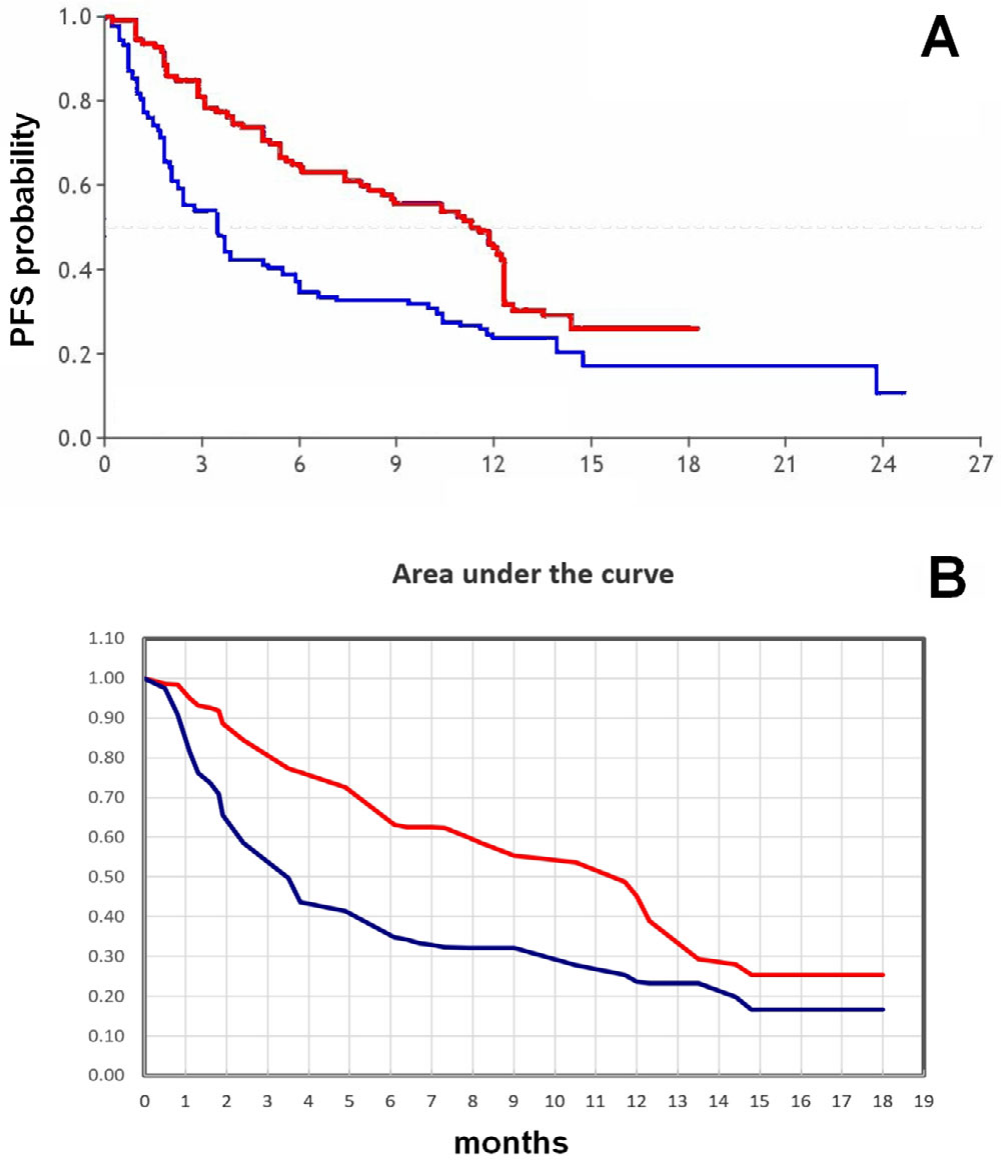
A, The values of RMST were estimated from these two Kaplan‐Meier curves for CART patients (n = 128; in red) and controls (n = 190; in blue). The original datasets were published by Jagannath et al. [1]. The figure shows the two curves uploaded onto the digitizing software. B, The area under the survival curve was separately estimated for CART patients (in red) and controls (in blue). Both curves were truncated (“restricted”) at the so‐called milestone at 18 months
